# Gastric Perforation Secondary to Fungal Gastritis in an Immuno-Competent Adult

**DOI:** 10.7759/cureus.13156

**Published:** 2021-02-05

**Authors:** Ankit Rai, Bhargav Gajula, Navin Kumar, Akanksha Malik

**Affiliations:** 1 General Surgery, All India Institute of Medical Sciences Rishikesh, Rishikesh, IND; 2 Pathology, National Institute of Pathology, New Delhi, IND; 3 Pathology, All India Institute of Medical Sciences Rishikesh, Rishikesh, IND

**Keywords:** mucormycosis induced gastric perforation, gastric perforation by fungal colonization, invasive fungal infections, gastric pathology

## Abstract

Gastrointestinal (GI) tract perforation is a surgical emergency. The epidemiology and etiology of perforation vary considerably across geography. Lower GI tract perforations in the elderly predominate in the West compared to upper GI perforations in the younger population in the tropics. Fungi and viruses have been reported to cause GI perforations in immuno-compromised individuals but it is rare in immuno-competent individuals. We report a very rare case of gastric perforation secondary to fungal gastritis in an immuno-competent 35-year-old female who presented with features of peritonitis. At emergency laparotomy, gastric perforation was found which was repaired by the Cellan-Jones method. Perforation edge biopsy findings were consistent with fungal etiology. She responded well to Antifungal therapy. We conclude that fungal etiology can be considered in patients with gastric perforation without any history of peptic ulcer disease (PUD) or use of oral non-steroidal anti-inflammatory drugs.

## Introduction

Peritonitis and the resultant sepsis and systemic complications due to perforation of the gastrointestinal (GI) tract are still responsible for significant mortality. Non-traumatic bowel perforation may occur due to a wide range of causes from immune-mediated conditions such as Crohn's disease, celiac sprue; infectious causes such as *Salmonella typhi*, *Mycobacterium tuberculosis*, *Ascaris lumbricoides*, *Entamoeba histolytica*; drugs such as non-steroidal anti-inflammatory drugs (NSAID) and steroids [1]. Site of perforation can provide a diagnostic clue in establishing an etiology, such as ileal perforation due to Salmonella and Crohn’s disease, jejunal perforation in celiac disease, or collagenous sprue, pre-pyloric or duodenal perforation in NSAID abusers, and *Helicobacter pylori* infection. Emergent surgical exploration is, however, required for the final diagnosis and definitive management [1]. Fungal and viral peritonitis in patients of GI tract perforation has been reported in immuno-compromised patients such as those with AIDS, malignancies, and individuals on systemic immuno-suppressive therapy [2]. Reports of fungal enteritis in immuno-competent individuals are rare. Fungal colonization of the stomach and gastritis impairs the process of gastric ulcer healing and increases the chance of ulcer-related complications. We present a case of non-traumatic gastric perforation secondary to fungal infection in an immuno-competent adult after written informed consent from the patient.

## Case presentation

A 35-year-old housewife, non-smoker, and without comorbidity, presented in the emergency department with complaints of sudden onset generalized pain in the abdomen with four episodes of non-bilious vomiting for two days. There was no associated history of peptic ulcer disease (PUD) or the use of non-steroidal anti-inflammatory drugs (NSAID) or any factor leading to an immunocompromised state like alcohol abuse, diabetes, and lymphoma, leukemia, renal disease, malnutrition, and long-term treatment with steroids and antibiotics. She was dehydrated, and her vital signs included a pulse rate of 108/min, blood pressure of 98/76 mm of Hg, respiratory rate of 22/min. Her temperature was recorded as 98.9 °F and SpO_2_ of 99% on room air. The abdominal examination revealed a distended abdomen with diffuse tenderness and rigidity and absent bowel sounds. The remainder of the physical examination was non-contributory.

Investigations

Initial laboratory tests showed a WBC count of 17,500/µL and Hb of 10.8 g/dL. Total protein and albumin were 4.1 g/dL and 2.2 g/dL, respectively, and the rest of the blood parameters were within normal limits. The patient was negative for HIV. Blood gas analysis was suggestive of compensated metabolic acidosis. X-ray chest showed free air under the right dome of the diaphragm.

Diagnosis

Because of the elicited history and clinical and radiological findings of the patient, the provisional diagnosis of hollow viscus perforation with peritonitis was made.

Treatment

**Figure 1:**
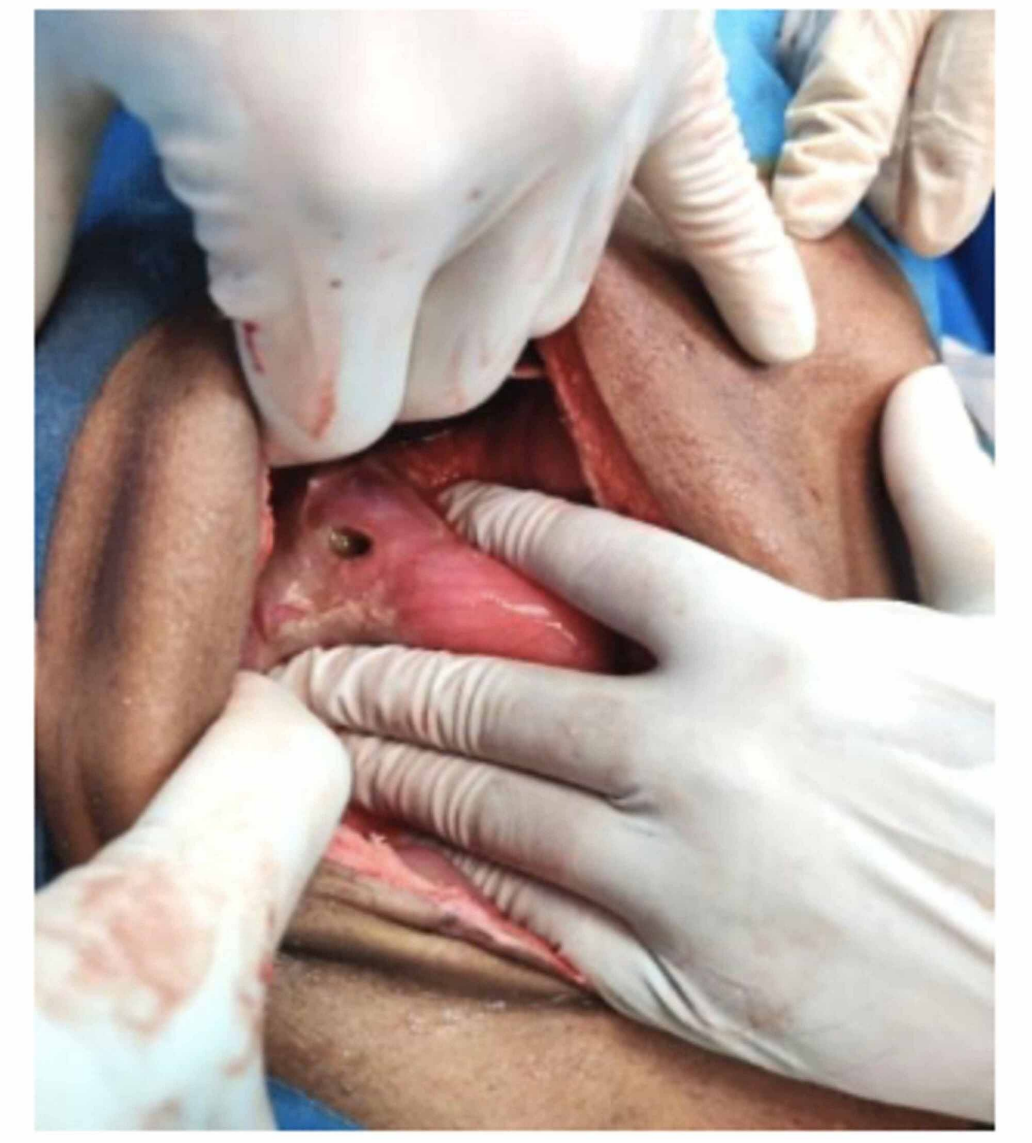
A 2 cm × 1 cm perforation over the anterior wall of the stomach close to the lesser curvature

**Figure 2:**
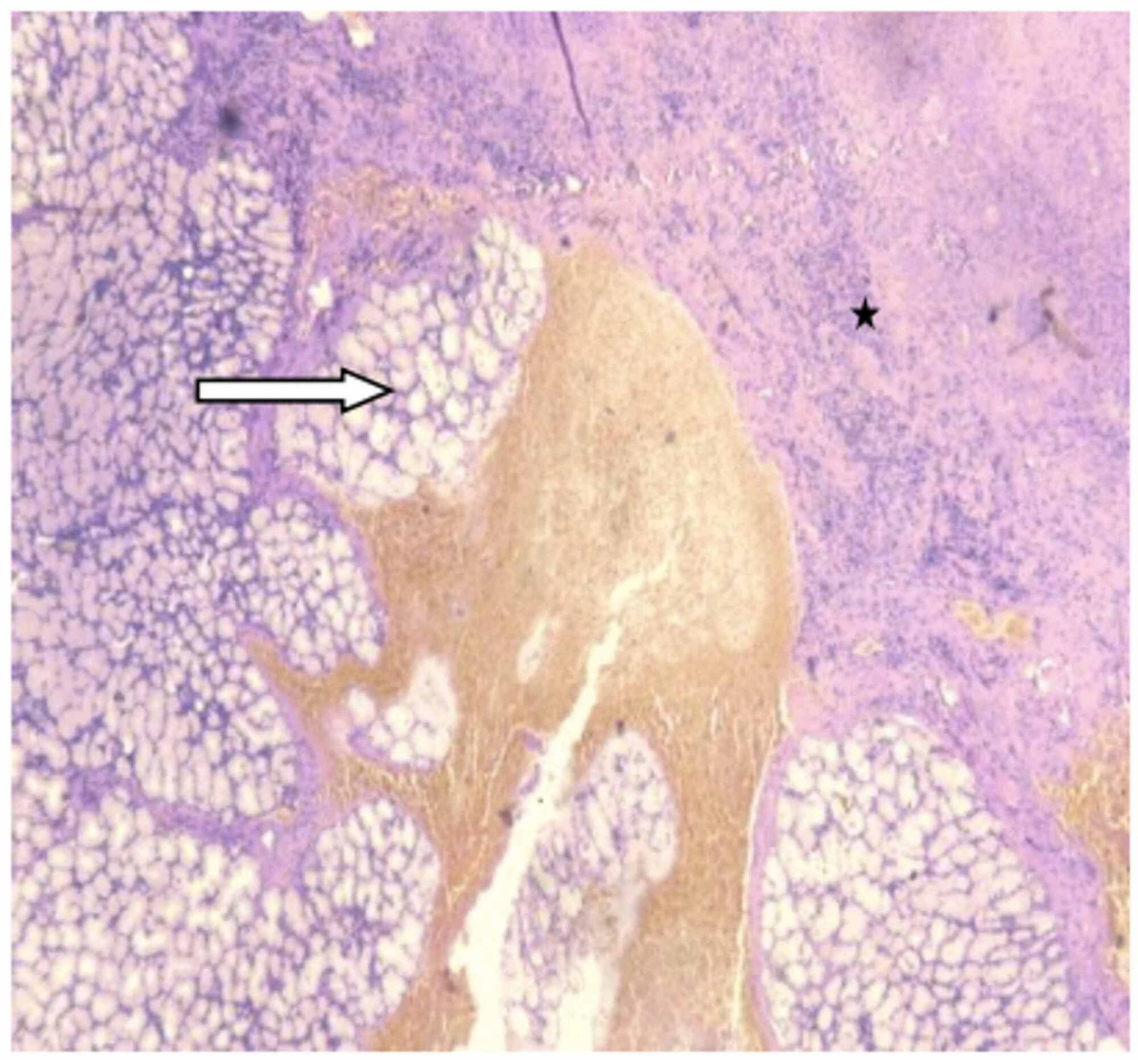
Microphotograph showing gastric glands (white arrow) with areas of hemorrhage and necrosis (H&E, 100x) H&E: hematoxylin and eosin.

**Figure 3:**
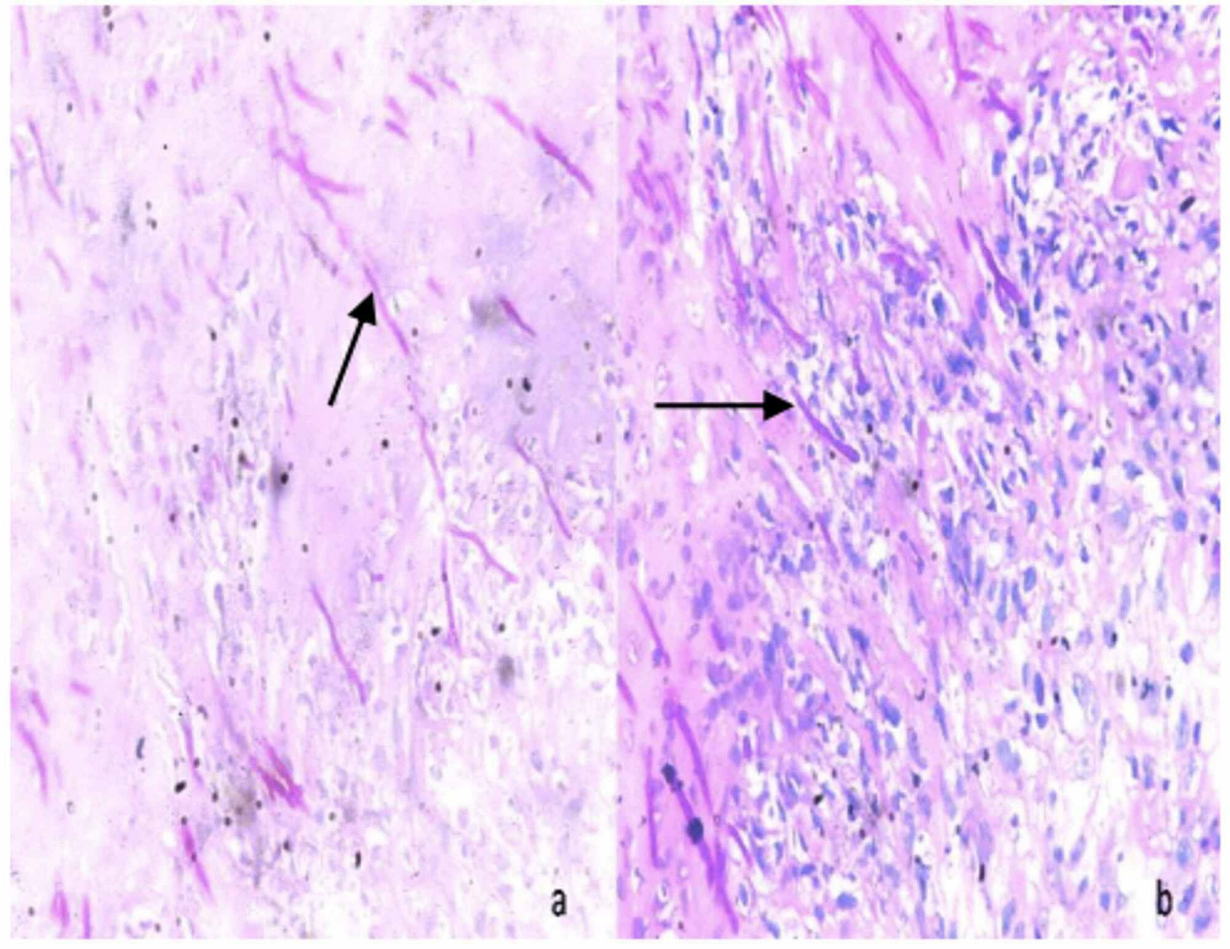
Microphotograph of histopathological examination of perforation edge with PAS and GMS staining showing thin filamentous, non-branching PAS-positive, diastase-resistant fungal organisms (arrow; PAS, 400x; PAS-D, 400x) PAS: periodic acid-Schiff, GMS: Grocott methenamine-silver.

The patient underwent exploratory laparotomy after written informed consent. Intra-operatively, 2 liter of biliopurulent peritoneal effluent was present with pus flakes over the stomach, small bowel, and omentum. A 2 × 1 cm^2^ perforation was noted in the anterior wall of the stomach (Figure 1). Pedicled omental patch (Cellan-Jones) repair of gastric perforation was done with Silk 2.0 sutures after thorough peritoneal lavage with normal saline. The patient was started empirically on injectable antibiotic Ceftriaxone 1 g and Metronidazole 500 mg intravenous 12 hourly & 8 hourly doses respectively for 5 days. Perforation edge biopsy showed fragments of gastric antral mucosa and surrounding fibro collagenous tissue with necrosis and acute on chronic inflammatory infiltrate. Few thin fungal hyphae, some with septae were noted in the fibro collagenous tissue and necrotic areas (Figure 2). PAS (Periodic acid-Schiff) and GMS (Grocott methenamine-silver) staining showed fungal organisms (Figure 3). Findings were consistent with mucormycosis as the cause of perforation. The peritoneal fluid culture was sterile. Antifungal therapy (Fluconazole) was added to the treatment regimen.

Outcome and follow-up

The patient's recovery was uneventful and discharged from the hospital on the sixth post-operative day. Oral antifungal Fluconazole 100 mg once daily for seven days was advised alongside proton pump inhibitor therapy for six weeks at discharge. At one month follow-up, the patient was doing fine with no complaints. Gastro-duodenoscopy in the follow-up period after six weeks of surgery showed complete healing of the ulcer.

## Discussion

Perforation peritonitis is the most common surgical emergency in India. Majority of these patients present with well-established generalized peritonitis with varying degrees of septicemia. There is a major difference in the epidemiology and etiology of GI tract perforations in this part of the world compared to the West [3]. The majority of the Indian patients are young adults, whereas the mean age of patients presenting in the west is between 45 and 60 years. Infections are the most common cause of perforation in India which include *H. pylori* infection, tuberculosis, and typhoid fever in the adult population [4]. Foreign body, ischemia, diverticula, and Crohn's disease and malignancy are common causes of perforation in the West [5].

Primary fungal infection of the GI tract is uncommon and accounts for only 7% of cases but is associated with 85% mortality [6]. The most common site of fungal GI tract infection is the stomach, followed by the colon and ileum, however, gastric perforation is a rare clinical presentation in such cases [7]. Mucormycosis is a rare and often fatal opportunistic infection in patients with diabetes mellitus, leukemia, lymphoma, HIV infection, or post-transplant patients on systemic immunosuppressants [6,8]. Salah et al. reported a case of gastric perforation related to mucormycosis in an immuno-compromised adult with heroin abuse, diabetes, hypertension, and chronic kidney disease on dialysis whereas Kyo et al. reported gastric perforation due to mucormycosis in a patient with acute myeloid leukemia [7,8]. Patricia et al. presented a case of GI bleeding with gastric perforation due to mucormycosis in an immuno-competent host [6].

GI mucormycosis can present with symptoms depending on the site involved. Nonspecific symptoms of abdominal pain, nausea, and vomiting are among the common symptoms. Because of the high mortality associated with GI tract mucormycosis, a high index of suspicion is necessary for immunocompromised patients for rapid diagnosis and prompt initiation of antifungal therapy. Mucormycosis in an immunocompetent host is rare and the occurrence of gastric mucormycosis is unusual [6]. There is an evidence-based guideline for the evaluation and treatment of invasive aspergillus infection. Direct microscopy, culture, and histopathological examination with or without PCR are recommended for diagnosis [9]. Polyene antifungals within one week of presentation have been associated with significantly improved survival [10]. There is a lack of high-level evidence regarding optimal antifungal therapy for mucormycosis. The role of combination therapy in mucormycosis is still not clear, although some retrospective data suggest that combining polyenes with echinocandins may be beneficial [11]. Discontinuation or dose-reduction of all immunosuppressive medications should also be considered in these patients.

## Conclusions

Fungal etiology is a very rare cause of gastric perforation in immuno-competent individuals and early diagnosis and treatment will reduce the significant morbidity and mortality. It should be considered in patients with gastric perforation without any history of PUD or NSAID and upon confirmation anti-fungal treatment should be started. Peritoneal fluid fungal cultures in GI tract perforations may be considered based on the suspected immune status of the patient. However, routine fungal cultures are not required. Fungal enteritis should prompt further evaluation to look for the cause of immuno-suppression such as an occult malignancy or an infection.
